# Somatostatin and Somatostatin-Containing Interneurons—From Plasticity to Pathology

**DOI:** 10.3390/biom12020312

**Published:** 2022-02-15

**Authors:** Monika Liguz-Lecznar, Grzegorz Dobrzanski, Malgorzata Kossut

**Affiliations:** Nencki Institute of Experimental Biology, 3 Pasteur Str., 02-093 Warsaw, Poland; g.dobrzanski@nencki.edu.pl

**Keywords:** somatostatin, plasticity, disease, somatostatin interneurons

## Abstract

Despite the obvious differences in the pathophysiology of distinct neuropsychiatric diseases or neurodegenerative disorders, some of them share some general but pivotal mechanisms, one of which is the disruption of excitation/inhibition balance. Such an imbalance can be generated by changes in the inhibitory system, very often mediated by somatostatin-containing interneurons (SOM-INs). In physiology, this group of inhibitory interneurons, as well as somatostatin itself, profoundly shapes the brain activity, thus influencing the behavior and plasticity; however, the changes in the number, density and activity of SOM-INs or levels of somatostatin are found throughout many neuropsychiatric and neurological conditions, both in patients and animal models. Here, we (1) briefly describe the brain somatostatinergic system, characterizing the neuropeptide somatostatin itself, its receptors and functions, as well the physiology and circuitry of SOM-INs; and (2) summarize the effects of the activity of somatostatin and SOM-INs in both physiological brain processes and pathological brain conditions, focusing primarily on learning-induced plasticity and encompassing selected neuropsychological and neurodegenerative disorders, respectively. The presented data indicate the somatostatinergic-system-mediated inhibition as a substantial factor in the mechanisms of neuroplasticity, often disrupted in a plethora of brain pathologies.

## 1. Somatostatin and SOM-INs—An Overview

Somatostatin (SOM) is a multi-functional peptide hormone produced by neurons and endocrine cells in the brain, gastrointestinal system, retina, immune and neuroendocrine cells, and pancreatic D cells of the islets of Langerhans, in response to different stimuli. This is a signaling molecule acting as an endogenous inhibitory regulator of different cellular functions, including development, proliferation, metabolism, secretion and neuromodulation. The D cells of the islets secrete SOM to inhibit the release of insulin and glucagon; in the hypothalamus, SOM inhibits the release of growth and thyroid-stimulating hormones from the anterior pituitary; in the gastrointestinal tract, it inhibits cholecystokinin, gastrin, secretin and vasoactive intestinal peptide (VIP) secretion, as well as the release of gastric acid [1]. In inflammatory cells such as lymphocytes, monocytes, or macrophages, SOM acts as a regulator of local immune responses exerting anti-inflammatory and antinociceptive effects, while, in the nervous system, it acts as a neurotransmitter and neuromodulator controlling the efficiency of synaptic transmission and regulating neuronal functions [2].

The first characterization of somatostatin by Brazeau et al. (1973) [3] came from a search for a growth-hormone-releasing factor, which resulted in the discovery of a 14-amino-acid peptide (SS-14) that inhibited the growth factor. Further studies revealed the existence of a diverse family of somatostatin peptide hormones displaying heterogeneity of amino acid composition and chain length [4]. This heterogeneity arises from multiple genes coding different peptide variants, precursor molecule processing differences and tissue-specific biosynthesis variations. Somatostatin is initially secreted as a 116-amino-acid precursor, preprosomatostatin, which undergoes endoproteolytic cleavage to prosomatostatin, which is further processed to the active form. In mammals, two active forms of different lengths of the amino acid chain, somatostatin-14 and somatostatin-28, are known. The half-life of endogenous somatostatin is 1–3 min and, as a polypeptide chain, it is primarily eliminated via metabolism through rapid degradation by the peptidase enzymes present in cells and plasma [5]. The somatostatinergic system also encompasses cortistatin, an evolutionary-related peptide derived from a distinct gene but sharing high structural and functional similarity with SOM [6,7]. However, due to the differences in tissue expression patterns, SOM and cortistatin can exhibit some functional differences. For example, cortistatin can enhance slow-wave activity and can consolidate short- and long-term memories [8].

Somatostatin actions are mediated by six subtypes of seven-transmembrane domain G-protein-coupled receptor, termed SSTR1-5 (with two splice variants of SSTR2: SSTR2A, SSTR2B), encoded by separate genes, binding endogenous somatostatin with low nanomolar affinity to initiate a broad spectrum of biological effects. All SSTRs share a common molecular topology, that is, amino terminus outside the cell, three extracellular loops, a hydrophobic core of transmembrane α-helices, three intracellular loops and a carboxyl terminus inside the cell. They are widely distributed throughout many tissues, ranging from the central nervous system to the pancreas and gut, pituitary, kidney, thyroid, lung and immune cells. SSTRs are overexpressed in some tumors and SSTRs imaging can help to identify a variety of neuroendocrine malignancies [9].

All SSTRs are Gi-protein-coupled receptors and, upon activation, they inhibit adenylyl cyclase, thus decreasing intracellular cyclic AMP and intracellular Ca^2+^. It is, however, worth stressing that some of them (SSTR2, SSTR3 and SSTR5) can also activate facilitatory G proteins which positively regulate protein phospholipase C and, as a result, activate protein kinase C [2]. The common pathway shared by SSTRs involves the activation of phosphotyrosine phosphatase (PTP) and modulation of mitogen-activated protein kinase (MAPK); however, each receptor subtype can be coupled to multiple intracellular transduction pathways and exerts many cellular consequences, including modulation of ion secretion, regulation of gene expression, cell proliferation, cell adhesion and apoptosis. Among all SSTRs, only SSTR5 has a higher binding affinity for SST-28, while others have a similar affinity for SST-14 and SST-28.

The first attempts of localizing somatostatin in the brain were undertaken in the mid-1970s [10]; however only in the mid-1980s, it was shown that somatostatin is present in a subset of GABAergic cells in the brain [11,12]. Nowadays, SOM is known to be a marker of the second largest of GABAergic interneurons [13]. In rodents, parvalbumin-containing, somatostatin-containing and serotonin receptor-5-HT3aR-containing cells form non-overlapping groups, essentially covering the entire population of cortical GABAergic interneurons. Somatostatin has a dense expression in the hippocampus, cortex, amygdala, hypothalamic nuclei, limbic and sensory system [14,15,16]. It influences different physiological processes, including locomotor activity [16] and sleep [17], but also higher brain functions such as emotions [18,19] and cognitive functions such as learning and memory [20,21].

In nerve cells, somatostatin is produced in the endoplasmic reticulum, stored in dense-core vesicles and is mainly released in a Ca^2+^-dependent manner [22]. Some experiments have shown, however, that other, Ca^2+^-independent, non-exocytotic ways of SOM release are also possible [23]. SOM can modulate neuronal activity via both pre- and post-synaptic mechanisms. The activation of most SSTRs (with the exception of SSTR3) in the brain leads to membrane hyperpolarization and inhibition of Ca^2+^ influx induced by depolarization [24]. In the hippocampus, exogenous SOM hyperpolarizes principal cells and, in addition, induces a presynaptic inhibition of excitatory synaptic transmission in those cells [25], while the injection of purified SOM into the primary visual cortex presynaptically suppresses and modulates excitatory neurotransmitter release to parvalbumin (PV) neurons [26]. The presynaptic inhibition of GABAergic synaptic transmission by SOM has also been reported in the hippocampus [27] and basal forebrain [28]. However, SOM action is not restricted to neurons since it was shown that SOM can strengthen astrocytic Ca^2+^ responses upon the successive activity of SOM-INs [29]. In vivo, SOM signaling is activated by different stressors, such as handling or immobilization [30,31,32], maternal separation [33], pain [31] and hypoxia [34], but also by exposure to a stressful challenge, such as elevated plus maze [35]. Moreover, SOM signaling is engaged during repetitive sensory stimuli [36], as well as learning and memory [37,38], since the blockade of SSTRs can impair, while SSTRs stimulation with agonists can improve sensory processing and support memory tasks (see [39]).

In nerve cells, somatostatin is considered a specific neuronal marker characteristic for the second-largest subpopulation of GABAergic interneurons (SOM-INs), as well as in the long-range GABAergic neurons [40]. Somatostatin and GABA are localized in the same neurons, they are released through a calcium-dependent mechanism and depend on action potential discharge. It is considered that the co-releasing of SOM and GABA can boost the inhibitory action of the classical GABA neurotransmitter [41,42,43]. As a neuropeptide SOM is stored in dense-core vesicles, which need a longer time and higher-frequency stimulation to release theirs content [44,45]. Therefore, SOM would act slower and with longer-lasting effects than GABA [46]. Their co-release results in facilitating the inhibitory action of the classical neurotransmitter GABA [42].

The dominant form of SOM-IN, the Martinotti cell, with low-threshold regular spiking properties and high spontaneous activity levels, both in the cerebral cortex and in the hippocampus, inhibits apical dendrites of pyramidal cells. Martinotti neurons regulate pyramidal cell activity via an extensive axonal arbor in layer 1 (L1) that forms synapses onto apical dendrites of pyramidal cells, modulating generation and synaptic integration [47]. In the cortex, they regulate converging cortico-cortical connections, conveying input both from the thalamus and from other cortical regions and L1 inputs from neuromodulatory innervation. Martinotti cells project densely to pyramidal cells located within a 200 μm radius [48] and are recruited in a feedforward manner by activated pyramidal neurons for which they provide feedback inhibition [49]. Silencing them with optogenetics results in increased activity and bursts the firing of pyramidal neurons. In this way, SOM-INs mediate top-down integration of behaviorally relevant information. Yuste proposed that dense connectivity of SOM-INs maintained a “blanket inhibition” over the cortex and holes in this blanket are caused by the action of interneurons containing the vasoactive intestinal peptide, which inhibits SOM-INs [48,50].

Non-Martinotti-type SOM-INs in mice are found in the somatosensory barrel cortex L4-5 and project mainly to L4 [51,52,53]. Morphologically, these cells are classified as basket cells, double-bouquet cells, or other shapes [40]. L5 non-Martinotti interneurons account for approximately one-third of all L5 SOM-INs and regulate the activity of the excitatory cells of L4, but not of L5 [54]. The non-Martinotti SOM-INs from cortical layer 4 regulate, by disinhibition, gating of thalamic input by inhibitory interneurons containing parvalbumin. The activation of SOM-INs by acetylcholine increases their inhibitory output on PV interneurons; as a result, PV-INs stop inhibiting pyramidal neurons so thalamocortical activation is facilitated [53].

A separate group of SOM neurons is long-range projecting GABAergic neurons, which connect cortical areas across the areal boundaries. They have been found mainly in cortical L6 and the white matter, but some fraction has also been localized in L 2/3. Since these neurons express a modulator of acetylcholine receptors (Lypd6), they can have the unique function of modulating cortical processing or rhythmic oscillatory activity through the convergence of GABAergic transmission and nicotinic signaling [55,56].

SOM-INs are connected with themselves and with other types of inhibitory interneurons by excitatory electrical synapses, facilitating widespread simultaneous inhibitory action. This characteristic makes them a likely candidate for participation in brain oscillations [57]. SOM-INs are essential in the propagation of low-frequency oscillations (theta band) [58] and cortical slow waves linked to NREM sleep [59]. It was recently demonstrated that SOM-INs are also the main contributors to the generation of slow-frequency gamma oscillations (30–60 Hz) in the cerebral cortex and the hippocampus [60]. Interestingly, although electrophysiological recordings did not find inhibitory synapses between SOM-INs, immunocytochemical investigations demonstrated the presence therein of all SSTRs subtypes [61].

Somatostatin regulates an extensive panel of endocrine and exocrine systems in the body and the disturbances of its secretion can have very severe consequences. The overproduction of SOM in the pancreas delta islet cells results in the development of a neuroendocrine tumor called somatostatinoma. On the other hand, SOM, due to its ability to downregulate the overproduction of specific hormones, is used to treat many neuroendocrine tumors, such as insulinoma or thyrotropinoma. However, due to the short half-life of endogenous somatostatin, synthetic analogs (lanreotide, octreotide, pasireotide) with higher stability are used for the treatment [62].

## 2. SOM INs in Learning-Induced Plasticity

### 2.1. Synaptic Plasticity

Long-term potentiation (LTP) is a widely investigated form of long-term plasticity being considered as a cellular mechanism that underlies learning and memory and is predominantly studied in the hippocampus [63]. The somatostatinergic system is suggested as an important modulator of LTP induction [64,65,66]. Exogenous somatostatin was shown to induce LTP at excitatory synapses onto hippocampal SOM-INs [64]. Such process was prevented by (1) SSTRs inhibition, (2) SOM depletion with cysteamine treatment and (3) GABA_A_ receptors’ blockade. Collectively, these results suggest that the endogenous somatostatin neuropeptide, via controlling GABA_A_ inhibition, plays a critical role in LTP induction at excitatory synapses onto SOM-INs [64]. The importance of the somatostatinergic system in LTP induction was also proved in another study, in which somatostatin gene knockout mice presented a decrease in CA1 principal cell’s LTP [65]. SOM can facilitate mossy fiber LTP in CA3 neurons [66] or dentate medial perforant path LTP [67], while SOM-INs are located in the CA1 oriens/alveus region and enhance LTP in the Schaffer collateral pathway [68]. Interestingly, although SOM enhances LTP at the Schaffer collateral synapses onto hippocampal CA1 apical and basal dendrites, the activation conditions of the somatostatin systems in these two compartments are different [69]. In the hippocampus, SOM appears to have different effects at different hippocampal synapses and those differences between hippocampal regions may be due to the distinct localization of SOM receptors in each region [70]. Moreover, SOM-INs specifically gate LTP in the basolateral amygdala afferents from the dorsomedial prefrontal cortex (dmPFC-BLA pathway) [71]. In the barrel cortex, L2/3 SOM-INs were found to be important elements of the disinhibitory circuit, responsible for LTP induction at intracortical synapses within L2/3 pyramidal neurons [72].

### 2.2. Plasticity in the Cerebral Cortex

The involvement of SOM-INs in learning attracts increasing attention as to their role in the formation of new neuronal circuits in the hippocampus, in the amygdala, in primary sensory cortices and the associative (primarily prefrontal) cortex. The most frequently considered action of a neuronal circuit is the influence of axons of L5 Martinotti neurons upon apical dendrite of L2/3 and L5 pyramids. SOM-INs axons form a dense plexus in L1 and may influence the dendritic spike of pyramids. Dendritic spikes lead to the burst firing of neurons, which is an important mechanism of LTP induction, i.e., synaptic plasticity. The inhibitory action of SOM-INs upon apical dendrites would negatively influence this process. This is well illustrated by the results obtained by Sheffield et al. (2017) from the hippocampus [73]. They demonstrated that the formation of hippocampal place cells occurs during transient periods of SOM-INs’ activity suppression when the dendritic spike can be triggered. The silencing of SOM-INs happens in new environments [73,74] when novelty prompts spatial learning. This suppression is likely due to the activation of VIP-INs by neuromodulatory or motor inputs [75,76]. VIP-INs strongly connect to SOM-INs and, in this way, the disinhibition of pyramidal neurons, which underlies an adult cortical plasticity in V1, may be achieved [77].

Inhibition of SOM-INs (disinhibition of pyramids) may also be due to the action of cortical L1 inhibitory interneurons. Abs et al. (2018) characterized a group of L1 inhibitory interneurons, which inhibit the distal dendrites of pyramids [78]. The activity of these neurons is blocked by the action of Martinotti SOM-INs.

The inhibitory action of SOM-INs on the apical dendrite can be linked to a selection of inputs onto pyramids. A single SOM-IN of L5 makes only a few synapses with the pyramid dendrite [79] and, during learning, dendritic disinhibition can be very selective and lead to the potentiation of specific inputs [80]. Experiments by Cichon and Gan (2015) showed that different learning tasks induced dendritic calcium spikes on different apical tuft branches of a single pyramid in the motor cortex [81]. This phenomenon depended on SOM-INs and was abolished when they were deleted or inactivated.

An interesting concept of SOM-INs’ function in learning was given by Adler et al. [38]. They stressed the importance of the stability of the activation pattern of pyramids arising during learning. During motor skill learning, in the primary motor cortex, they observed an increased sequential firing of defined groups of pyramids in layer 2/3. At the same time, SOM-INs increased or decreased their activity. External manipulation of SOM-INs activity during training had a detrimental effect on this stability of the pyramid firing sequence and impaired the memory trace, which implies that SOM-INs modulate the neuronal ensemble forming during motor-skill learning.

Interestingly, during learning, SOM-INs activity is different from the activity of other interneurons. Observations of networks formed by inhibitory interneurons with 2-photon calcium imaging during learning visual discrimination found that SOM-INs increase selectivity for task-related stimuli [82]. Moreover, the SOM-IN network loses its correlation with the pyramidal network. It means that its activity ceases to appear at the same time as that of the pyramidal network, creating conditions more permissive for synaptic plasticity and learning [83]. In this way, SOM-INs gate learning-related plasticity by preventing the top-down and bottom-up information stream inputs from coinciding [82]. The same paper described increased selectivity for discrimination in both SOM and PV interneurons, disproving the frequent assumption that inhibitory interneurons are not plastic.

A circuit in layer 4 described by Xu et al. [53], in which L4 non-Martinotti SOM cells weakly inhibit L4 excitatory cells and strongly inhibit PV-INs, in the barrel cortex, is involved in fear learning when stimulation of whiskers is used as the conditioned stimulus. Dobrzanski et al. (2021) [84] found that the chemogenetic inhibition of SOM-INs of L4 impaired both learning and learning-induced plasticity of the barrel cortex.

The SOM-INs in the association cortices can also be modified by learning. Interestingly, the proportion of SOM-INs in the prefrontal cortex is higher than that in sensory cortices [85]. Cummings and Clem (2020) [86] found that, in prelimbic cortex neurons, after fear learning, synaptic transmission was potentiated in L2/3 SOM-INs, with a higher frequency of EPSC, increased glutamate release on SOM-INs and potentiation of excitatory synapses on SOM-INs. Inhibiting SOM-INs during learning prevented electrophysiological changes and decreased freezing.

The role of SOM-INs in working memory was demonstrated by Kim et al. [37]. They found that, during the delay period of a spatial memory task, the SOM-INs of the medial prefrontal cortex showed strong persistent activity, which is a marker of neurons participating in working memory.

The activation of SOM-INs in non-sensory cortical areas can modulate intracortical long-range projections and affect signal processing circuits in primary sensory cortices. For example, the optogenetic activation of orbitofrontal cortex projection to V1 reduces the amplitude of responses via recruitment of local SOM-INs [87]. The influence of attention, emotion, mood, pain and reward are important in learning and can impact the formation of neuronal ensembles underlying memory trace. An interesting example of long-range coordination being controlled by SOM-INs comes from the paper by Abbas et al. (2018) [88]. They studied the role of prefrontal cortex SOM-INs in a spatial working memory task. During training, optogenetic inactivation of SOM-INs was achieved with optrodes, which simultaneously recorded neuronal activation. Local field potentials were registered from mPFC and hippocampus. SOM-INs inhibition in mPFC during encoding phase impaired working memory. Moreover, it interfered with the long-range synchrony of neuronal activations between mPFC and hippocampus, indicating that SOM-INs may facilitate long-range synchrony. Recent results revealed that the long-range intracortical inputs to SOM-INs are weak [89,90]. However, VIP-INs are strongly activated and, as they project to SOM-INs, a disinhibitory effect, causing activation of pyramidal neurons, was observed [89].

### 2.3. Plasticity in the Hippocampus

The hippocampus is a key structure in episodic memory, associative learning and spatial navigation [91,92]. One of the most widely used models to study hippocampus-dependent memories is contextual and trace fear conditioning [93]. The hippocampus integrates environmental cues with aversive stimuli, forming a neural representation of the context that predicts danger [94]. The activity of CA1 pyramidal neurons is modulated by oriens lacunosum-moleculare (OLM) SOM-INs that provide strong inhibition onto distal parts of pyramidal apical dendrites, thus gating the flow of information from CA3 and entorhinal cortex into CA1 [95,96]. CA1 SOM-INs are active during classical conditioning, as shown with calcium imaging [97], and their intrinsic excitability is increased after learning [98]. In an animal model of Alzheimer’s disease, OLM SOM-INs synapses were affected by decreased cholinergic input and the process contributed to memory impairment [99]. Indeed, OLM SOM-INs receive cholinergic inputs from the medial septum [100] and acetylcholine-expressing neurons are responsive to aversive stimuli and drive SOM-INs activity during fear learning [97]. Temporary somatostatin depletion after cysteamine administration or somatostatin knock-out in transgenic mice impairs contextual-dependent, but not cue-dependent fear conditioning [65]. SOM-INs are crucial in contextual fear learning and their optogenetic inactivation increases the activity of CA1 pyramidal cells and prevents fear learning [97]. On the synaptic level, one pivotal factor that influences long-term synaptic plasticity of hippocampal excitatory neurons, the basis for learning and memory, is the Mechanistic Target of Rapamycin associated with protein Complex 1 (mTORC1) [101]. mTORC1 was also found to be a critical element of contextual-fear-learning-induced plasticity of hippocampal SOM-INs; it was proved that a loss of mTORC1 activity, selectively in SOM-INs, disrupted long-term contextual fear learning and mTORC1 activity is needed for fear-conditioning-induced long-term potentiation of excitatory inputs synapses onto SOM-INs [102]. In another hippocampus-dependent learning paradigm, a directed spatial navigation task in virtual reality, CA1 SOM-INs activity was suppressed during exposure to a novel environment, but it recovered when animals learned to navigate in it, as shown with calcium imaging [74].

SOM-INs of other than CA1 regions of hippocampus or hippocampus-related structures were also shown to be essential in learning. Stefanelli et al. (2015) [103], using c-fos brain mapping and opto- and chemo-genetic techniques, have shown that increased activity of dentate gyrus SOM-INs during contextual fear conditioning and spatial recognition tasks influenced the size of memory representations by the dendritic lateral inhibition of granule cells. Somatostatin-expressing interneurons excited by nearby activated granule cells can inhibit other granule neurons, controlling the size of the activated neuronal population, thus providing a selectivity of memory engrams. With calcium imaging, Besnard et al. (2019) [104] discovered that a stable population of SOM-INs of the dorsolateral septum (a bridge structure between the hippocampus and subcortical areas that is engaged in linking contextual information with action) was responsive to foot shock during contextual fear learning and their optogenetic manipulations alter conditioned fear responses, so they are hypothesized to scale fear-learning responses.

Altogether, an increasing number of data suggests that SOM-INs are critical components of hippocampal learning-related circuits. By receiving neuromodulatory inputs from subcortical stress- and aversive-related areas, they pose to control memory formation and engram size, as well as shaping the behavioral expression of learned associations.

## 3. Somatostatin and SOM-INs in Neuropathology

Reduced SOM expression or SOM-IN number is the hallmark of many neurological disorders, including Alzheimer’s, Parkinson’s and Huntington’s disease, schizophrenia and depression. The mentioned disorders are all associated with mood-related symptoms and/or altered cognitive functions in which SOM and SOM-INs are deeply engaged. Therefore, the low performance of the somatostatinergic system can influence several biological functions, which can be implicated in the complex mechanisms of brain disorders [105]. Alterations in the somatostatinergic system, resulting in the deficits of inhibition and, eventually, an imbalance between excitation and inhibition, can induce negative adaptive changes, leading to decreased plasticity. Reduced plasticity, in turn, prevents effective compensation, causing further dysfunction of synapses and, finally, pathological neurochemical and neurophysiological processes directing towards diseases.

Identifying the mechanisms and somatostatin-targeting interventions may represent a new avenue for treatments However, the usage of SOM analogs in the mentioned diseases is hindered by their limited ability to penetrate the brain [39].

### 3.1. Major Depression

The levels of somatostatin and GABA in cerebrospinal fluid are often reduced in patients with major depression [106] and they recover when MDD symptoms subside. Post mortem studies found decreased levels of somatostatin mRNA expression in the prefrontal cortex, anterior cingulate and amygdala [107,108,109], while histopathological and immunocytochemical studies found a reduced number of somatostatin-positive cells in the amygdala [110]. Interestingly, both cerebrospinal fluid SOM levels and the somatostatin neurons’ number were more affected in females [111]. Animal studies support the hypothesis that disrupted SOM interneuron function may contribute to mood disorder symptoms. In mice, chronic stress, which is a major risk factor for major depression, induced lowered mRNA levels of SOM and the GABA-synthesizing enzyme Gad67 in the cingulate cortex and reduced SOM interneuron but not pyramidal neuron mRNA expression [112,113]. It has to be stressed that the expression of SOM is regulated by the level of brain-derived neurotrophic facor (BDNF) and mice with knockout (KO) of somatostatin gene showed elevated corticosterone, reduced expression of BDNF and Gad67 expression and elevated anxiety-/depressive-like behaviors [112]. The manipulation of SOM-INs in the prefrontal cortex showed their influence on behavior. Chemogenetic or optogenetic SOM-IN silencing elevated depressive behaviors [18]. Conversely, genetic disinhibition had antidepressant-like effects [114] and so did corticolimbic infusion of exogenous SOM peptides [115,116].

Investigations of the role of SOM receptors in the hippocampus of mice revealed that SSTR2 agonists selectively produced anxiolytic-like behaviors, whereas both SSTR2 and SSTR4 agonists had antidepressant-like effects. Moreover, SSTR2 receptors were sensitive to antidepressant treatment—their changed activity was observed during imipramine administration in the chronic mild stress model of depression [117]. In SST2KO mice, high corticosterone levels and anxiety were found and depressive-like behaviors were observed in both SST2KO and SST4KO strains [116]. Sibille posited that low SOM levels have a causal role in depression, as it is associated with molecular (reduced gene expression of BDNF, Corticostatin and Gad67), neuroendocrine (high corticosterone) and behavioral manifestation of human depression [112]. However, his recent work on the chemogenetic inhibition of SOM-INs found that allosteric modulators of 5alpha-GABA_A_ receptors ameliorated the depressive signals, thus confirming the role of both GABAergic and somatostatinergic transmission in major depression [118].

### 3.2. Schizophrenia

GABAergic deficits in schizophrenia have been documented in many studies and those deficits were especially pronounced in SOM interneurons [119]. Initially, the changes in GABAergic neurons were described for the dorsolateral prefrontal cortex (DLPFC), but, later, they were also found in many cortical areas, including the primary sensory and motor cortex [120]. In DLPFC, Morris et al. (2008) [121] confirmed a lower level of SOM transcript in post mortem samples from schizophrenic patients, detected by in situ hybridization studies. They observed that the number of neurons expressing somatostatin mRNA was reduced by 26% in cortical L4 and by 23% in L5. The decline in the number of SOM neurons was found in post mortem samples of the hippocampus, in a stereological study—55% fewer SOM-INs were found in CA1 [122]. Wang et al. (2011) [123] found reductions in SOM cells density in the parasubiculum, presubiculum and entorhinal cortex. These deficits can result in network desynchronization and affect cortico-hippocampal integration.

The role of SOM neurons in behavioral changes, characteristic of schizophrenia, was investigated by several groups. Abbas et al. [88] focused on the enhancement of oscillatory synchrony between mPFC and hippocampus in the theta and gamma range during working memory, a function impaired in schizophrenia. They optogenetically inhibited prefrontal PV- or SOM-INs in the mPFC of mice performing a spatial working memory task. It was found that the inhibition of SOM- but not PV-INs during the memory encoding phase impaired spatial memory. The impairment was associated with decreased theta synchrony between hippocampus and mPFC and worse spatial encoding in mPFC.

Adams et al. (2020) [124] examined frontotemporal oscillations in memory. With magnetoencephalography recordings, they found that subjects with schizophrenia exhibited decreased mPFC–mTL (medial temporal lobe) theta phase coupling during cued spatial memory retrieval and were impaired during spatial memory. Moreover, the mTL region showing reduced theta phase coupling coincided with areas of reduced density of SOM-INs, as revealed by PET examination.

Hamm and Yuste (2016) [125] suggested a causal link in schizophrenia between SOM interneurons impairments, low-frequency population oscillatory dynamics and deficits in salience processing in patients. In a study on the animal schizophrenia model, they examined the influence of SOM interneurons deficiency on the electroencephalographic phenomenon called decreased mismatch negativity, found in schizophrenic patients. Mismatch negativity consists of amplified cortical potential response to a deviant stimulus. Their study showed that chemogenetic suppression of SOM-INs specifically eliminated deviance detection. SOM-INs generated population-level synchrony in the theta and beta bands and, in their experiments, Hamm and Yuste showed a deficit in oscillatory activity in the theta band in mice with silenced SOM-INs in the visual cortex [125].

### 3.3. Epilepsy

The role of somatostatin in epilepsy was suggested by Vezzani and Hoyer (1999) [126] because somatostatin is released during seizures [127]. Moreover, mRNA, peptide and receptors for SOM change their level following seizures [128]. The first data showing epilepsy-related neuronal loss of SOM-INs from the hilus of the dentate gyrus of the hippocampus were reported by Sloviter (1987) [129] in rats and confirmed in humans [130]. This loss may impair feedback inhibition of dentate granular neurons [131] and was found to reduce spontaneous inhibitory postsynaptic currents and monosynaptic inhibitory postsynaptic potentials by about 40% [132]. The loss of SOM-INs was not observed exclusively in the hilus of the dentate gyrus but also in the CA1 and CA3 after intrahippocampal injection of the kainic acid; interestingly, in this study, similar changes were not reported for calcium-binding proteins such as parvalbumin, calbindin, or calretinin [133].

SOM receptors are also affected by epilepsy. It was shown in patients that SSTR2 mRNA is strongly upregulated in the dentate gyrus; however, mRNA and immunoreactivity for this receptor were decreased in CA1 and CA3 [134]. Vezzani and Hoyer [126] showed that the injection of the octapeptide, which is an analog of somatostatin and SSTR2 agonist, reduced the number and duration of seizures in rats. SOM application also decreases epileptic seizures. Mazarati and Telegdy (1992) [135] found that SOM infused into ventricles, hippocampus, or amygdala could significantly reduce the seizure severity. According to Tallent [131], this action in CA1 is due to a decrease in excitatory postsynaptic currents evoked by stimulating Schaeffer collaterals [25] and, in CA3, by the presynaptic inhibition of glutamate release at associational/commissural synapses [136].

Another possible way neuropeptide SOM may reduce epileptogenic plasticity was suggested by Baratta et al. [70], who found that the SOM level modulates synaptic plasticity at lateral perforant path synapses (an epileptogenic locus), preventing the generation of long-term potentiation by the post-synaptic inhibition of N-type Ca^2+^ channels, which hampers the depolarization of the synaptic membrane [70]. Interestingly, the anti-seizure stimulation of cholinergic neurons in the septum was found, in a mouse model, to be due to the cholinergic excitation of hippocampal SOM interneurons [137].

### 3.4. Alzheimer’s Disease

Changes in somatostatin content in the cerebral cortex, especially in the temporal lobe, are a hallmark of Alzheimer’s disease [138] and a decrease in the number of SOM-INs was observed in temporal lobe brain samples from patients [139,140]. In the hippocampus, the number of SOM-expressing cells was reduced by 50% [141] and SOM receptors were also reduced in the temporal cortex of patients with AD [142]. In murine models of Alzheimer’s disease in which protein deposits built up, atrophy and synaptic SOM-INs abnormalities have been observed [99,143]. The direct involvement of SOM in Alzheimer’s disease is suggested by the fact that, in amyloid precursor protein transgenic mice, SOM has been shown to increase the activity of neprilysin, an enzyme that promotes Aβ degradation. Moreover, SOM knockout mice presented a 50% decrease in neprilysin activity and an increase in Aβ42 accumulation [39].

Cognitive symptoms in Alzheimer’s disease are, among others, the result of oscillatory activity altered by amyloid deposits and hypersynchrony of neural circuits [144]. The deposits can impair the induction of gamma-dependent long-term synaptic enhancement, important in spatial memory [145]. The optogenetic activation of somatostatin or parvalbumin interneurons in the hippocampus of mice injected with amyloid-beta oligomer restored theta and gamma oscillations and the related synaptic plasticity [146]. Experiments by Huang et al. (2020) [58] showed a specificity of action of these two types of interneurons—SOM-IN activity resynchronized relative to theta oscillations and PV-INs, relative to gamma oscillations. Moreover, the transplantation of progenitor cells overexpressing the Nav1.1 sodium channel subunit, characteristic of somatostatin and parvalbumin interneurons, improved cognitive function and oscillatory properties in a mouse model of disease [147].

## 4. Conclusions

Inhibitory neurons, including SOM-INs, have a high controlling activity in the brain, perhaps best described for PN-INs. However, SOM-INs are not only inhibitory neurons; they are also a source of a hormone of critical importance, influencing many endo/exocrine systems in the body. Somatostatin action in the brain is far from being completely understood and neither is the characteristic distribution of its receptor subtypes. In this review, the pleiotropic actions that somatostatin and SOM-INs play in the nervous system are briefly summarized. Molecular and biochemical alterations of this system can result in precisely localized inhibition and modification of synaptic plasticity. SOM-INs can also, being linked by electrical synapses, propagate their signals at a larger scale and increase or decrease the balance between excitation and inhibition within a cortical region. Deficits in their number or function influence brain oscillation patterns and may induce deregulation of excitatory cell input/output, initiating a cascade of negative adaptive changes, which, together with decreased plasticity and impaired learning abilities, may cause further dysfunctions, leading to pathological neurochemical and neurophysiological processes directing towards diseases.

**Scheme 1:**
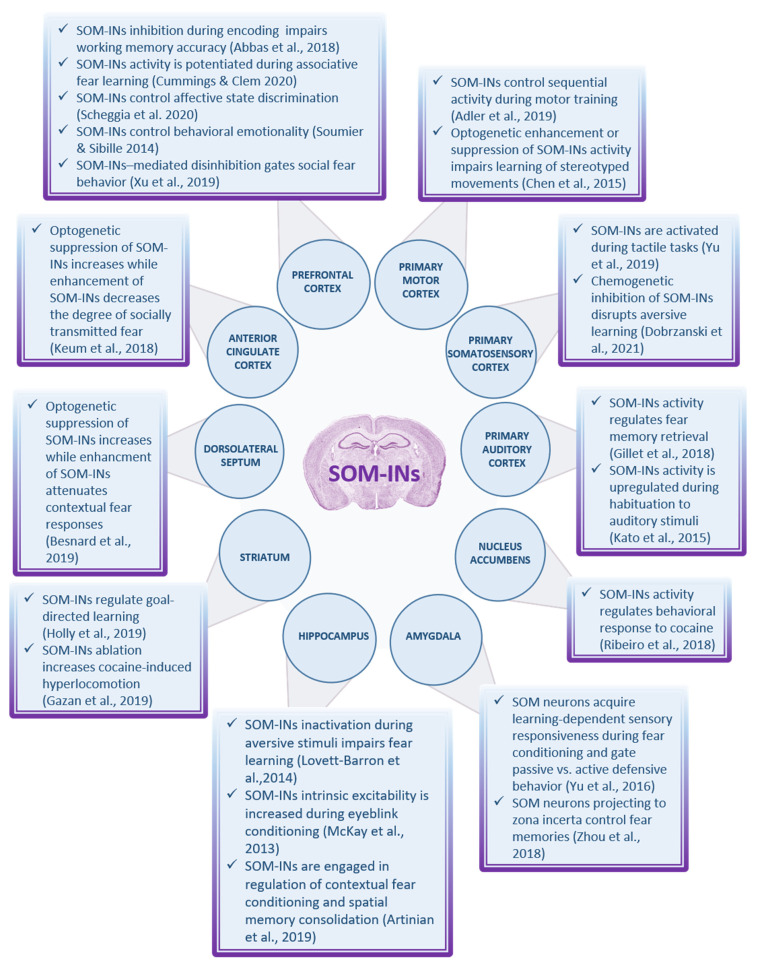
SOM-INs across the brain in learning and memory—recent advances [18,36,38,76,84,86,88,97,98,102,104,148,149,150,151,152,153,154,155,156,157].

The main recent findings regarding the role of somatostatin and SOM-INs in proper brain functioning as well in pathologies were characterized here. To extend information in the manuscript, the recent advances picturing the role of SOM-INs in learning and memory, as well the involvement of the somatostatinergic system in neurodegenerative disorders were introduced (Scheme 1 and Table 1, respectively).

## Figures and Tables

**Table 1:** Somatostatin and somatostatin-expressing interneurons in major neurodegenerative disorders—the advances.

DISORDER	DESCRIPTION	REFERENCES
ALZHEIMER’S DISEASE (AD)	Selective loss of SOM-INs in the temporal cortex of AD patients	[140]
	Selective loss of SOM-INs in mice model of AD	[139]
	SOM-IN-involved synaptic and circuit dysfunction in mice with AD-pathology	[99,146,158]
	Transplantation of Nav1.1-overexpressing interneurons improved cognitive functioning in transgenic mice model of AD	[147]
	Optogenetic activation of SOM-INs restored brain functions impaired by amyloid β oligomers	[146]
PARKINSON’S DISEASE (PD)	Decreased expression of SOM in GABAergic interneurons derived from induced pluripotent stem cells of patients with *parkin* mutations	[159]
	In in vivo optogenetic activation of the primary motor cortex, SOM-INs alleviated motor symptoms in mouse and rat models of PD	[160,161]
HUNTINGTON’S DISEASE (HD)	Mice with Somatostatin Receptor 1 and 5 Double Knockout induced neurochemical changes in Huntington’s Disease	[162]
	Loss-of-huntingtin function during the development led to deficits in forebrain SOM-INs, which may contribute to the symptoms of HD in adulthood	[163]
	Optogenetic modulation of SOM-INs as a tool for modeling of HD	[164,165]
AMYOTROPHIC LATERAL SCLEROSIS (ALS)/FRONTOTEMPORAL DEMENTIA (FTD)	Hyperactive SOM-INs contributed to excitotoxicity of layer 5 primary motor cortex pyramidal neurons in mice models of ALS and FTD. The excitotoxicity could be reversed with selective SOM-INs’ ablation.	[166]
